# Fetal Huanjiang mini-pigs exhibit differences in nutrient composition according to body weight and gestational period

**DOI:** 10.1371/journal.pone.0199939

**Published:** 2018-07-13

**Authors:** Qian Zhu, Peifeng Xie, Huawei Li, Cui Ma, Wanghong Zhang, Yulong Yin, Xiangfeng Kong

**Affiliations:** 1 Hunan Provincial Key Laboratory of Animal Nutritional Physiology and Metabolic Process, Key Laboratory of Agro-ecological Processes in Subtropical Region, National Engineering Laboratory for Pollution Control and Waste Utilization in Livestock and Poultry Production, Institute of Subtropical Agriculture, Chinese Academy of Sciences, Changsha, China; 2 University of Chinese Academy of Sciences, Beijing, China; 3 Research Center of Mini-Pig, Huanjiang Observation and Research Station for Karst Ecosystems, Chinese Academy of Sciences, Huanjiang, China; University of Illinois, UNITED STATES

## Abstract

Low birth weight may negatively affect energy storage and nutrient metabolism, and impair fetal growth and development. We analyzed effects of body weight (BW) and gestational period on nutrient composition in fetal Huanjiang mini-pigs. Fetuses with the lowest BW (LBW), middle BW (MBW), and highest BW (HBW) were collected at days 45, 75, and 110 of gestation. Crude protein (CP), crude fat, amino acid (AA), and fatty acid (FA) concentrations were determined. The BW gain, carcass weight, fat percentage, and uterus weight of sows increased as gestation progressed, as did litter weight, average individual fetal weight, fetal body weight, and dry matter (DM). The concentrations of Ala, Arg, crude fat, Gly, Pro, Tyr, C14:0, C16:0, C16:1, C18:1n9c, C18:2n6c, C18:3n3, C18:3n6, C20:0, C20:3n6, saturated FA (SFA), and monounsaturated FA (MUFA) increased significantly as gestation progressed. The percentage of skeleton, and the ratio of the liver, lung, and stomach to BW decreased as gestation progressed. There were also significant reductions in the concentrations of CP, Asp, Glu, His, Ile, Leu, Lys, Phe, Ser, Thr, essential AA (EAA), acidic AA, C17:0, C20:4n6, C22:6n3, unsaturated FA (UFA), polyunsaturated FA (PUFA), n-3PUFA, n-6PUFA as gestation progressed, and reductions in EAA/total AA (TAA), PUFA/SFA, and n-3/n-6 PUFA. The LBW fetuses exhibited the lowest BW and crude fat, C14:0, C16:1, C17:0, C18:2n6c, and MUFA concentrations at days 75 and 110 of gestation. They also exhibited lower Tyr concentration at day 45 of gestation and lower Glu concentration at day 75 of gestation than HBW fetuses. These findings suggest that LBW fetuses exhibit lower amounts of crude fat and several FAs during mid-gestation and late-gestation, which may in turn affect adaptability, growth, and development.

## Introduction

In swine production facilities, approximately 15–20% of newborn piglets exhibit low birth weight [1, 2]. This low birth weight may lead to lower rates of survival, slower postnatal growth, suboptimal carcass quality, lower efficiency of nutrient utilization, and an increase in the number of days required to reach the common slaughter weight [3–5]. Low birth weight is caused by intrauterine growth retardation (IUGR) during gestation [6]. In general, fetal body weight (BW) that is more than two standard deviations below the mean BW at the corresponding gestational age is considered indicative of IUGR [7, 8]. Pigs are multifetal domestic animals and frequently exhibit IUGR.

Fetal growth is affected by the nutritional, metabolic, and endocrine status of the maternal system [9]. Maternal under-nutrition or over-nutrition may result in IUGR [1] that negatively affects the metabolism, growth, and development of fetal pigs, as well as numerous metabolic pathways and their feeding efficiency and disease susceptibility [10]. Despite advances in nutrition and management techniques, low birth weight and substantial litter variation in fetal weight frequently occur during the late gestational phase, and the precise mechanisms underlying fetal pig development remain to be fully elucidated.

Huanjiang mini-pigs are anatomically and physiologically similar to humans, and their relatively small size makes them easier to handle than other varieties of pigs [11–13]. In addition, they are the most popular local pigs in Guangxi, China. Dietary nutrients play important roles during gestation, and the feeding management and feed intake can affect maternal reproductive performance and fetal growth and development [14]. Huanjiang mini-pigs are usually grazed and fed diets with lower nutrient level or with imbalanced nutrition, and have lower body weight at the first service and bigger litter size. Therefore, these various characteristics of Huanjiang mini-pigs render them more susceptible to low birth weight. Therefore, Huanjiang mini-pigs were considered an appropriate experimental model.

The growth rates of fetal pigs vary over the course of gestation, accelerating during the second half of pregnancy [15]. As the body composition of fetal pigs may vary in conjunction with growth rates, the present study aimed to analyze nutrient composition in fetal Huanjiang mini-pigs in terms of BW and gestational period. We also aimed to determine whether fetal pigs with different BWs exhibited differences in body composition, as well as the time at which these potential differences occurred, in order to provide a theoretical basis for developing nutritional interventions targeting fetuses with low birth weight.

## Materials and methods

All pigs used in the present study were managed in accordance with the Chinese Guidelines for Animal Welfare. The experimental design and procedures were reviewed and approved by the Animal Care and Use Committee of the Institute of Subtropical Agriculture, Chinese Academy of Science, China.

### Animals, diets, and treatments

The present study was conducted at the Mini-pig Research Center at the Huanjiang Observation and Research Station for Karst Ecosystems in Huanjiang, Guangxi, China. A total of 24 primiparous Huanjiang mini-pigs with an initial BW of approximately 30 kg were obtained from a mini-pig farm located in Jixiang, Huanjiang, Guangxi Province, China (108°27'40.8" E, 25°9'50" N) and reared in eight pens, with three mini-pigs per pen. The animals were fed a diet formulated in accordance with the recommendations of the Chinese National Feeding Standard for Swine (Table 1), which is commonly used in commercial Huanjiang mini-pig farms. Animals were allowed access to water *ad libitum* for the duration of the experiment. The animals were fed at 8:00, 15:00, and 18:00 each day, and the quantity of each feed was approximately 2% of maternal BW.

**Table 1:** Ingredient and nutrient levels of the basal diet (air-dry basis).

Dietary ingredient	Rate (%)	Nutrient	Level^2^
Corn	54.0	Digestive energy (MJ/Kg)	13.40
Soya meal	12.0	Crude protein (%)	12.04
Rice bran	30.0	Ca (%)	0.78
Premix^1^	4.0	P (%)	0.62
Total	100.0	Lysine (%)	0.53
		Arginine (%)	0.65
		Proline (%)	0.67

^1^ Provided by per kg premix: VA 301 000 IU, VD 52 800 IU, VE 742 IU, VK_3_ 71 mg, VB_1_ 30 mg, VB_2_ 177 mg, VB_6_ 32 mg, VB_12_ 0.8 mg, nicotinic acid 1073 mg, D-pantothenic acid 540 mg, folic acid 22 mg, biotin 3.0 mg, chorine 8.0 g, Fe 2.0 g, Cu 1.0 g, Zn 3.5 g, Mn 1.3 g, I 14 mg, Co 35 mg, Se 8.3 mg, Ca 200 mg, P 20 mg;

^2^ The values of nutrient levels were analyzed.

### Sample collection

Sows were fasted and weighed at days 45, 75, and 110 of gestation. One sow per pen was randomly selected and sacrificed under commercial conditions via electrical stunning (120 V, 200 Hz) and exsanguination [16], after which each sow was dissected, and its uterus and fetuses were each individually weighed. The size of each fetus was recorded. The carcass, muscle, fat, skeleton, liver, lungs, and stomach were dissected and weighed to determine the percentages of live BW (tissue weight [kg]/BW [kg] × 100%) or ratio to live BW (organ weight [g]/BW [kg]) that they comprised. Fetuses with the lowest, middle (similar to mean BW), and highest BW (LBW, MBW, and HBW, respectively) in each litter were dissected and their internal organs were removed. The remaining fetuses were stored in sealed plastic bags at -80°C prior to further analysis.

### Determination of dry matter, crude protein, and crude fat

The fetal pigs were minced after weighing and dried in a vacuum-freeze dryer (CHRIST RVC2-25 CDPIUS; Christ Company, Osterode, Germany) in order to calculate the dry matter (DM) concentration. The crude protein (CP) concentration (N × 6.25) was determined using the Kjeldahl method in accordance with standards provided by the Association of Official Analytical Chemists [17]. The crude fat concentration was determined using the Soxhlet extraction method. Petroleum ether was used as the binary extracting solution [18].

### Determination of hydrolyzed amino acids

Approximately 0.1000 g of freeze-dried fetal pig powder was hydrolyzed in 10 mL of 6 mol/L hydrochloric acid solution at 110^°^C for 24 h. The suspension was diluted to 100 mL in double-distilled water [19], then 1 mL of supernatant was transferred to a 1.5-mL centrifuge tube and evaporated to dryness in a water bath at 65°C. The sample was then dissolved using 1 mL of 0.02 mol/L hydrochloric acid solution and filtered through a 0.45-μm membrane prior to analysis [20] with an ion-exchange AA analyzer (L8800, Hitachi, Tokyo, Japan).

### Determination of fatty acids

Different fatty acids (FAs) were identified via gas-liquid chromatography (7890A, Agilent, California, USA) of methyl esters as previously described [21, 22]. The FA composition was expressed as g/100 g of total identified FA. The following parameters were calculated based on the FA composition: the sum of saturated FA (SFA, C14:0 + C16:0 + C17:0 + C18:0 + C20:0); unsaturated FA (UFA, C16:1 + C18:1n9c + C18:1n9t + C18:2n6c + C18:3n3 + C18:3n6 + C20:1 + C20:3n6 + C20:4n6 + C22:6n3); monounsaturated FA (MUFA, C16:1 + C18:1n9c + C18:1n9t + C20:1); polyunsaturated FA (PUFA, C18:2n6c + C18:3n3 + C18:3n6 + C20:3n6 + C20:4n6 + C22:6n3); n-3PUFA (C18:3n3 + C18:3n6 + C22:6n3); n-6 PUFA (C18:2n6c + C20:3n6 + C20:4n6); and the ratios of PUFA to SFA and n-6 to n-3 PUFA.

### Statistical analysis

Sow data were analyzed via one-way analysis of variance. Fetal data were analyzed using a mixed-effects model, and the means were separated using Tukey’s method. Analyses were performed using SAS version 9.2 (SAS Institute, Inc., Cary, NC, USA). Results are presented as means and standard errors of the mean. Gestational periods, BWs, and their interactions were included in the statistical model. The level of statistical significance was set at *P* < 0.05. Probability values between 0.05 and 0.10 were considered indicative of trends.

## Results

### Reproductive performance of sows

The reproductive performance of sows is presented in Table 2. Uterus weight, litter weight, and average individual fetal weight increased (*P* < 0.05) as gestation progressed.

**Table 2:** The reproductive performance of sows at different gestation period (n = 8).

Items	Day 45 of gestation	Day 75 of gestation	Day 110 of gestation	SEM	*P-*values
Uterus weight (kg)	2.12	4.72	5.95	0.40	<0.0001
Fetus number	7.13	7.88	7.43	0.51	0.7754
Litter weight (g)	200.30	1455.40	3674.20	9.63	<0.0001
Average individual fetal weight (g)	25.59	183.40	507.30	3.28	<0.0001

### Body composition and organ ratio of sows

The body composition and organ ratios of sows are shown in Table 3. As gestation progressed, both BW and carcass weight increased (*P* < 0.05), and fat percentage tended to increase (*P* = 0.074). Skeleton percentage and the ratio of the liver, lung, and stomach to total BW decreased (*P* < 0.05), and muscle percentage tended to decrease (*P* = 0.070).

**Table 3:** The body composition and organ percentage of sows at different gestation period (n = 8).

Items	Day 45 of gestation	Day 75 of gestation	Day 110 of gestation	SEM	*P-*values
Body weight gain (kg)	21.11	28.42	41.53	0.76	<0.0001
Carcass weight (kg)	35.13	37.73	46.17	0.85	0.004
Muscle percentage	13.27	13.49	11.85	0.42	0.0701
Fat percentage	8.20	8.33	9.73	0.41	0.0736
Skeletal percentage	5.24	3.69	3.74	0.23	<0.0001
Liver ratio (g/kg)	20.66	16.96	14.31	0.51	<0.0001
Lung ratio (g/kg)	7.09	5.59	5.29	0.39	0.021
Stomach ratio (g/kg)	11.81	10.26	9.30	0.40	0.0033

### Body weight of fetal pigs

Table 4 presents the fetal BWs, which increased as gestation progressed (*P* < 0.05). The BWs of HBW fetuses were significantly higher than those of LBW fetuses at day 110 of gestation (*P* < 0.05), but not at days 45 or 75 of gestation. A trend (*P* = 0.072) was observed with regard to interaction between gestation period and fetal BW.

**Table 4:** Body weight and routine nutrient concentrations of fetal pigs with LBW, MBW, and HBW at different gestation period (n = 8).

Items	Day 45 of gestation			Day 75 of gestation			Day 110 of gestation			SEM	*P-*values		
	LBW^1^	MBW^2^	HBW^3^	LBW	MBW	HBW	LBW	MBW	HBW		Gestation period	Body weight	GP*BW
Body weight (g)	16.54^d^	19.75^d^	21.59^d^	152.21^c^	182.72^c^	209.51^c^	427.21^b^	513.00^a^^b^	575.03^a^	2.92	<0.0001	0.003	0.072
Dry matter (%)	9.78^c^	9.79^c^	9.97^c^	12.27^b^	11.91^b^	12.61^b^	18.73^a^	19.27^a^	19.22^a^	0.24	<0.0001	0.04	0.08
Crude protein (%)	13.72^ab^	14.15^a^	14.28^a^	13.00^b^^c^^d^	13.29^b^^c^	12.55^d^^c^^e^	12.27^d^^e^	12.41^d^^e^	11.93^e^	0.25	<0.0001	0.04	0.03
Crude fat (%)	2.63^d^	4.21^d^^c^	2.99^d^	3.33^d^	3.11^d^	7.55^a^	2.93^d^	5.31^b^^c^	6.05^a^^b^	0.37	<0.0001	<0.0001	<0.0001

^1^ LBW, the lowest body weight;

^2^ MBW, the middle body weight;

^3^ HBW, the highest body weight;

^a-e^ Values within a row without a common superscript letter differ (*P* < 0.05).

### Dry matter, crude protein, and crude fat concentrations of fetal pigs

The DM, CP, and crude fat concentrations of fetal pigs are shown in Table 4. DM and crude fat concentrations increased (*P* < 0.05) in fetuses with LBW, MBW, and HBW as gestation progressed, while CP concentration in fetuses with MBW and HBW decreased (*P* < 0.05). No significant differences (*P* > 0.05) in DM or CP concentrations were observed in fetuses with LBW, MBW, or HBW at any of the three different gestation periods. In addition, no significant differences in crude fat concentration were observed at day 45 of gestation. At day 75 of gestation, crude fat concentration was higher (*P* < 0.05) in HBW fetuses than in MBW or LBW fetuses. At day 110 of gestation, crude fat concentration in MBW and HBW fetuses was higher (*P* < 0.05) than that of LBW fetuses. Interaction effects (*P* < 0.05) were observed between gestation period and BW with regard to DM, CP, and crude fat concentrations.

### Amino acid composition of fetal pigs

The amino acid (AA) composition of fetal pigs is shown in Table 5. As gestation progressed, the Ala, Arg, Gly, Pro, and Tyr concentrations of fetuses with LBW, MBW, and HBW increased (*P* < 0.05), while the concentrations of Asp, Glu, His, Ile, Leu, Lys, Phe, Ser, Thr, and essential AA (EAA) decreased (*P* < 0.05). We also observed a significant decrease in the ratio of EAA/(total AA) TAA. Significant decreases (*P* < 0.05) in TAA concentration were observed in MBW and LBW fetuses as gestation progressed. The Cys and Val concentrations in fetuses with LBW, MBW, and HBW tended to decrease and then increase (*P* < 0.05) as gestation progressed, and a similar pattern was observed for TAA and non-essential AA (NEAA) in HBW fetuses.

**Table 5:** Composition of hydrolyzed amino acids of fetal pigs with LBW, MBW, and HBW at different gestation period (g/100g; n = 8).

Items	Day 45 of gestation			Day 75 of gestation			Day 110 of gestation			SEM	*P-*values		
	LBW^1^	MBW^2^	HBW^3^	LBW	MBW	HBW	LBW	MBW	HBW		Gestation period	Body weight	GP*BW
Arg	2.64^c^	2.68^c^	2.61^c^	2.71^b^^c^	2.70^b^^c^	2.54^c^	3.00^a^^b^	3.11^a^	3.13^a^	0.15	<0.0001	0.24	0.62
His	1.33^a^^b^	1.38^a^	1.30^a^^b^^c^	1.18^b^^c^^d^	1.18^b^^c^^d^	1.10^d^	1.13^c^^d^	1.13^c^^d^	1.14^c^^d^	0.11	<0.0001	0.24	0.62
Ile	1.67^a^	1.61^a^	1.57^a^^b^	1.45^b^^c^	1.46^b^^c^	1.37^c^	1.34^c^	1.36^c^	1.34^c^	0.11	<0.0001	0.07	0.51
Leu	3.73^a^	3.72^a^	3.58^a^	3.14^b^^c^	3.17^b^	3.00^b^^c^^d^	2.79^d^	2.86^b^^c^^d^	2.84^c^^d^	0.16	<0.0001	0.17	0.58
Lys	3.59^a^	3.52^a^	3.40^a^^b^	3.18^b^^c^	3.20^b^^c^	3.00^c^	3.06^c^	3.02^c^	3.00^c^	0.16	<0.0001	0.03	0.71
Met	0.96	0.93	0.79	0.85	0.90	0.81	0.84	0.80	0.79	0.14	0.20	0.11	0.62
Phe	1.84^a^	1.87^a^	1.81^a^	1.59^b^	1.59^b^	1.50^b^	1.46^b^	1.50^b^	1.49^b^	0.12	<0.0001	0.32	0.70
Thr	1.98^a^^b^	2.01^a^	1.93^a^^b^	1.77^b^^c^	1.77^b^^c^	1.66^c^	1.65^c^	1.63^c^	1.63^c^	0.14	<0.0001	0.23	0.85
Val	2.21^a^	2.21	2.13^a^^b^	1.89^b^^c^	1.89^b^^c^	1.79^c^	2.14^a^^b^	2.02^a^^b^^c^	2.01^a^^b^^c^	0.15	<0.0001	0.14	0.86
EAA^4^	19.61^a^	19.68^a^	18.83^a^^b^	17.77^b^^c^^d^	18.13^b^^c^	16.70^d^	17.49^c^^d^	16.76^d^	17.36^c^^d^	0.32	<0.0001	0.013	0.015
Ala	2.73^a^^b^^c^	2.69^b^^c^	2.60^c^	2.61^c^	2.64^c^	2.48^c^	2.97^a^^b^	3.01^a^	3.00^a^^b^	0.16	<0.0001	0.27	0.69
Asp	3.94^a^^b^	4.00^a^	3.84^a^^b^^c^	3.59^a^^b^^c^	3.68^a^^b^^c^	3.45^c^	3.54^b^^c^	3.59^a^^b^^c^	3.56^b^^c^	0.18	<0.0001	0.21	0.87
Cys	0.65^a^	0.59^a^^b^	0.60^a^^b^	0.39^a^^b^^c^	0.25^c^	0.36^b^^c^	0.64^a^	0.49^a^^b^^c^	0.58^a^^b^	0.14	<0.0001	0.06	0.89
Gly	3.62^b^^c^	3.58^b^^c^	3.48^c^	3.96^b^	3.99^b^	3.88^b^^c^	4.79^a^	5.12^a^	5.21^a^	0.19	<0.0001	0.46	0.09
Glu	8.14^a^	8.03^a^	7.70^a^^b^	7.07^b^^c^	6.72^d^^c^	6.19^d^^e^	6.18^d^^e^	6.10^d^^e^	5.84^e^	0.26	<0.0001	0.0025	0.68
Pro	2.63^a^^b^	2.59^a^^b^	2.47^b^	2.85^a^^b^	2.86^a^^b^	2.74^a^^b^	3.24^a^	3.24^a^	2.85^a^^b^	0.23	0.0002	0.15	0.86
Tyr	0.93^a^^b^^c^	0.57^c^^d^	0.49^d^	1.17^a^	1.04^a^^b^	0.93^a^^b^^c^	0.79^b^^c^^d^	0.87^a^^b^^c^	0.85^a^^b^^c^^d^	0.17	<0.0001	0.009	0.03
Ser	2.21^a^^b^	2.25^a^	2.12^a^^b^^c^	1.92^c^^d^	1.94^b^^c^^d^	1.81^d^	1.90^c^^d^	1.90^c^^d^	1.91^c^^d^	0.15	<0.0001	0.26	0.78
NEAA^5^	24.88^a^	24.09^a^^b^	23.30^b^	23.08^b^	23.59^b^	21.70^c^	23.92^a^^b^	24.16^a^^b^	23.80^b^	0.35	<0.0001	0.0004	0.0524
TAA	44.50^a^	43.84^a^^b^	41.98^a^^b^^c^	40.85^c^^d^	41.58^b^^c^	38.34^d^	41.79^b^^c^	40.51^c^^d^	41.64^b^^c^	0.46	<0.0001	0.0016	0.0075
EAA/TAA	0.45^a^	0.45^a^	0.45^a^	0.43^b^	0.43^b^	0.43^b^	0.41^c^	0.41^c^	0.42^c^	0.027	<0.0001	0.31	0.65

^1^ LBW, the lowest body weight;

^2^ MBW, the middle body weight;

^3^ HBW, the highest body weight;

^4^ EAA: essential amino acids = Arg + His + Ile + Leu + Lys + Met + Phe + Thr + Val;

^5^ NEAA: nonessential amino acids = Ala + Asp + Cys + Gly + Glu + Pro + Tyr + Ser;

^a-e^ Values within a row without a common superscript letter differ (*P* < 0.05).

Relative to levels observed in HBW fetuses, Tyr and NEAA concentrations were higher (*P* < 0.05) in LBW fetuses at day 45 of gestation, while Glu concentration was higher at day 75 of gestation. The TAA and EAA concentrations were also higher (*P* < 0.05) in MBW fetuses at day 75 of gestation, while NEAA concentration was higher (*P* < 0.05) in MBW and LBW fetuses at days 75 and 110 of gestation, relative to levels observed in HBW fetuses.

Significant effects (*P* < 0.05) of gestation period × BW were observed on Tyr, EAA, and TAA concentrations in fetal pigs. A similar trend (*P* = 0.09) was observed for gestation period × BW interaction on Gly concentration.

### Fatty acid composition of fetal pigs

The FA composition of fetal pigs is shown in Table 6. As gestation progressed, significant increases (*P* < 0.05) in C14:0, C16:0, C16:1, C18:1n9c, C18:2n6c, C18:3n3, C18:3n6, C20:0, C20:3n6, SFA, and MUFA concentrations were observed in fetuses with LBW, MBW, and HBW, and significant decreases in C17:0, C20:4n6, C22:6n3, UFA, PUFA, n-3PUFA, and n-6PUFA concentrations were observed, along with corresponding significant decreases in PUFA/SFA and n-3/n-6PUFA (*P* < 0.05). C18:0, C18:1n9t, and C20:1 concentrations first decreased and then increased (*P* < 0.05) in fetuses with LBW, MBW, and HBW as gestation progressed.

**Table 6:** Composition of fatty acid of fetal pigs with LBW, MBW, and HBW at different gestation period (%; n = 8).

Items	Day 45 of gestation			Day 75 of gestation			Day 110 of gestation			SEM	*P-*values		
	LBW^1^	MBW^2^	HBW^3^	LBW	MBW	HBW	LBW	MBW	HBW		Gestation period	Body weight	GP*BW
C14:0	1.86^e^	1.99^e^	1.92^e^	2.22^d^	2.50^c^	2.38^c^^d^	2.73^b^	2.95^a^	3.11^a^	0.13	<0.0001	<0.0001	0.0038
C16:0	28.08^c^	28.10^c^	27.85^c^	29.40^b^	29.34^b^	29.06^b^	33.98^a^	34.00^a^	33.86^a^	0.23	<0.0001	0.10	0.97
C16:1	3.34^e^	3.38^e^	3.28^e^	4.36^d^	5.12b^c^	4.69^c^^d^	5.46^b^	6.17^a^	6.53^a^	0.20	<0.0001	<0.0001	<0.0001
C17:0	3.91^a^	3.95^a^	3.98^a^	2.42^c^	2.85^b^	2.79^b^	1.61^d^	1.63^d^	1.70^d^	0.03	<0.0001	0.0053	0.025
C18:0	18.11^a^	17.87^a^^b^	17.96^a^^b^	17.10^c^^d^	16.54^d^	17.26^b^^c^	18.16^a^	17.67^a^^b^^c^	17.35^b^^c^	0.24	<0.0001	0.0051	0.0095
C18:1n9c	18.72^c^^d^	18.44^d^	18.88^c^^d^	19.04^c^^d^	19.37^a^^b^^c^	19.35^b^^c^	20.02^a^^b^	20.06^a^^b^	20.20^a^	0.26	<0.0001	0.32	0.58
C18:1n9t	0.24^a^^b^^c^	0.25^a^^b^	0.25^a^^b^	0.19^b^^c^	0.16^c^	0.16^c^	0.26^a^^b^	0.22^a^^b^^c^	0.29^a^	0.08	<0.0001	0.44	0.16
C18:2n6c	2.80^e^	2.71^e^	2.99^e^	3.77^d^	3.96^d^	3.96^d^	4.46^c^	4.89^b^	5.44^a^	0.16	<0.0001	<0.0001	<0.0001
C18:3n3	0.27^c^	0.29^b^^c^	0.28^c^	0.35^b^	0.36^b^	0.35^b^	0.48^a^	0.45^a^	0.49^a^	0.08	<0.0001	0.85	0.40
C18:3n6	0.15^d^	0.17^c^^d^	0.16^c^^d^	0.25^b^	0.26^b^	0.24^b^^c^	0.32^a^^b^	0.29^a^^b^	0.35^a^	0.08	<0.0001	0.73	0.24
C20:0	0.12^d^	0.12^c^^d^	0.11^d^	0.18^b^^c^	0.17^b^^c^^d^	0.18^b^	0.44^a^	0.42^a^	0.46^a^	0.07	<0.0001	0.33	0.38
C20:1	0.57^a^^b^	0.51^a^^b^^c^^d^	0.53^a^^b^^c^^d^	0.50^b^^c^^d^	0.46^c^^d^	0.45^d^	0.60^a^	0.55^a^^b^^c^	0.57^a^^b^	0.09	<0.0001	0.0202	0.98
C20:3n6	0.66^b^	0.72^a^^b^	0.72^a^^b^	0.75^a^^b^	0.75^a^^b^	0.77^a^^b^	0.80^a^	0.83^a^	0.78^a^	0.10	<0.0001	0.35	0.50
C20:4n6	16.54^a^	16.93^a^	16.87^a^	15.51^b^	15.19^b^^c^	14.53^c^	8.72^d^	8.46^d^	7.04^e^	0.27	<0.0001	<0.0001	0.0002
C22:6n3	4.58^a^	4.64^a^	4.44^a^	3.69^b^	3.27^b^^c^	3.22^c^	1.84^d^	1.50^d^	0.99^e^	0.18	<0.0001	<0.0001	0.003
MUFA^4^	22.98^d^	22.54^d^	23.02^d^	24.21^c^	24.99^c^	24.54^c^	26.28^b^	26.65^a^^b^	27.52^a^	0.29	<0.0001	0.024	0.008
PUFA^5^	24.77^a^^b^	25.43^a^	25.17^a^	24.30^a^^b^	23.76^b^^c^	22.80^c^	17.07^d^	16.77^d^	15.09^e^	0.31	<0.0001	<0.0001	0.0008
PUFA/SFA	0.47^a^	0.49^a^	0.49^a^	0.47^a^	0.46^a^	0.46^a^	0.30^b^	0.30^b^	0.28^b^	0.05	<0.0001	0.34	0.09
SFA^6^	52.29^b^	51.72^b^	51.55^b^	51.69^b^	51.17^b^	51.50^b^	57.20^a^	56.85^a^	56.64^a^	0.31	<0.0001	0.041	0.78
UFA^7^	47.71^a^	48.28^a^	48.45^a^	48.31^a^	48.83^a^	48.50^a^	43.34^b^	43.15^b^	43.36^b^	0.32	<0.0001	0.30	0.48
n-3 PUFA^8^	5.01^a^^b^	5.08^a^	4.65^b^	4.22^c^	3.89^c^	3.79^c^	2.65^d^	2.23^d^^e^	1.99^e^	0.18	<0.0001	<0.0001	0.072
n-6 PUFA^9^	19.86^a^	20.51^a^	20.60^a^	20.11^a^	19.89^a^	19.85^a^	14.35^b^	14.48^b^	13.57^b^	0.31	<0.0001	0.40	0.045
n-3/n-6 PUFA	0.24^a^	0.25^a^	0.24^a^^b^	0.21^b^^c^	0.19^c^^d^	0.19^c^^d^	0.18^d^	0.15^e^	0.15^e^	0.05	<0.0001	0.0006	0.061

^1^ LBW, the lowest body weight;

^2^ MBW, the middle body weight;

^3^ HBW, the highest body weight;

^4^ MUFA = C16:1 + C18:1n9c + C18:1n9t + C20:1;

^5^ PUFA = C18:2n6c + C18:3n3 + C18:3n6 + C20:3n6 + C20:4n6 + C22:6n3;

^6^ SFA = C14:0 + C16:0 + C17:0 + C18:0 + C20:0;

^7^ UFA = C16:1 + C18:1n9c + C18:1n9t + C18:2n6c + C18:3n3 + C18:3n6 + C20:1 + C20:3n6 + C20:4n6 + C22:6n3;

^8^ n-3 PUFA = C18:3n3 + C18:3n6 + C22:6n3;

^9^ n-6 PUFA = C18:2n6c + C20:3n6 + C20:4n6;

^a-e^ Values within a row without a common superscript letter differ (*P* < 0.05).

At day 45 of gestation, n-3PUFA concentration was higher (*P* < 0.05) in MBW fetuses than in HBW fetuses. At day 75 of gestation, C14:0 and C16:0 concentrations were higher (*P* < 0.05) in MBW fetuses than in LBW fetuses. In addition, C17:0 concentration was higher (*P* < 0.05) in MBW and HBW fetuses, while C20:4n6, C20:6n3, and PUFA concentrations were lower (*P* < 0.05) in HBW fetuses than in LBW fetuses. C18:0 concentration was higher (*P* < 0.05) in HBW fetuses than in MBW fetuses at day 75 of gestation. At day 110 of gestation, C18:0 and n-3PUFA concentrations were higher (*P* < 0.05) in LBW fetuses than in HBW fetuses. The concentrations of C20:4n6, C20:6n3, and PUFA were higher (*P* < 0.05) in MBW and LBW fetuses, while MUFA concentration was lower (*P* < 0.05) than that of HBW fetuses. C14:0 and C16:1 concentrations were higher (*P* < 0.05) in MBW and HBW fetuses, while the n-3/n-6PUFA ratio was lower (*P* < 0.05) than that of LBW fetuses. C18:2n6c concentration was lower (*P* < 0.05) in LBW fetuses than it was in both MBW and HBW fetuses.

Significant interaction effects (*P* < 0.05) were observed between gestation period and BW with regard to C14:0, C16:1, C17:0, C18:0, C18:2n6c, C20:4n6, C22:6n3, MUFA, PUFA, and n-6PUFA concentrations. Trend-level interaction effects of gestation period × BW were observed on n-3PUFA (*P* = 0.072), PUFA/SFA (*P* = 0.09), and n-3/n-6PUFA (*P* = 0.061).

## Discussion

The present study aimed to determine the changes in the nutrient composition of fetuses according to BW and gestation period using Huanjiang mini-pig models. LBW fetuses exhibited lower amounts of crude fat and several FAs at mid-gestation and late-gestation, which may in turn affect adaptability, growth, and development.

During gestation, sows undergo dramatic changes and must acquire enough nutrition to maintain their own health and development and that of their fetuses. An improper supply of nutrition will negatively affect the health of sows as well as fetal growth and development [23]. In the present study, uterus weight, litter weight, and average individual fetal weights increased as gestation progressed, mainly due to continuous growth and development of the fetuses. However, no significant difference in fetus number was observed between days 45, 75, and 110 of gestation. This is likely because of the 20–45% of conceptuses lost throughout gestation, most are lost between 12 and 30 days after conception [24].

Maximal fetal growth occurs during the third trimester of pregnancy, during which sows must increase their nutrient intake to meet the demands of the growing fetuses [25]. Therefore, changes in the body composition of sows may reflect the utilization of the body’s nutrient reserves. In the present study, the BW gain and carcass weight of sows increased as gestation progressed, due to the continued growth of the mother and her conceptuses, and previous studies have indicated that maternal fluid expansion throughout pregnancy contributes to such increases [26]. We also observed that body fat percentage tended to increase as gestation progressed, which is beneficial to fetal growth. The percentage of muscle and the ratio of liver, lung, and stomach to BW decreased as gestation progressed, due to the rapid increase in the size of the conceptuses.

Fetal growth and development is influenced by several factors including genetics, epigenetics, maternal maturity, maternal nutrition during gestation, uterine capacity, placental efficiency, litter size, day of gestation, and other environmental factors [8, 27, 28]. Our findings indicate that the BW of fetal pigs undergoes dynamic changes as gestation progresses, suggesting that nutrients are progressively accumulated in fetal pigs. A previous study demonstrated that the BW of fetal pigs increases sharply as gestation progresses, and that 90% of fetal growth occurs during the late stage of gestation [29]. At day 110 of gestation, the BW of HBW fetuses was significantly higher than that of LBW fetuses in the present study, while no significant difference in BW was observed at days 45 and 75 of gestation, suggesting that the within-litter variation of fetal BW becomes more apparent during the late stage of gestation, in accordance with the findings of previous studies [23, 30]. Such findings also indicate that fetal growth retardation occurs principally during the late gestational stage [23, 31].

The nutrient composition of fetal pigs reflects nutritional deposition in the maternal uterus. In the present study, we observed that DM and crude fat concentrations in fetuses increased as gestation progressed, regardless of fetal BW. These findings are consistent with those of previous studies in which DM, CP, and crude fat concentrations increased exponentially in fetal pigs as gestation progressed [15, 29]. In the current study, CP concentration in fetuses with LBW, MBW, and HBW decreased as gestation progressed, possibly due to the use of different body tissues when determining CP. McPherson et al. [29] demonstrated that CP concentration in both fetal carcass and brain decreased as gestation progressed, further suggesting that the rate of CP deposition decreases in the late stage of gestation. At days 75 and 110 of gestation, crude fat concentration in HBW fetuses was significantly higher than that of LBW fetuses, suggesting that distinctions in crude fat concentration occur among fetal pigs of different BWs during mid-gestation and late-gestation, and that HBW fetuses can accumulate more fat than LBW fetuses. Fat concentration is related to energy storage in the body; therefore, our findings suggest that HBW fetuses conserve more energy and are more capable of adapting to the postnatal environment than LBW fetuses.

AAs have extremely different biochemical properties and functions, playing a prominent role not only as building blocks for proteins but also as substrates for the synthesis of a range of physiologically important molecules of immense biological importance [32–34]. Furthermore, AAs are known to exert various effects on body composition, blood flow, metabolic regulation, growth, and development [9]. Several studies have indicated that the concentrations of AAs vary remarkably in fetal fluid during pregnancy [35–37]. An insufficient supply of AAs from the mother to the fetus may result in IUGR [9, 38]. In the present study, Asp, Glu, His, Ile, Leu, Lys, Phe, Ser, Thr, and EAA concentrations, as well as EAA/TAA, decreased as gestation progressed, suggesting that the accretion of these AAs in fetal pigs progressively decreases (*i*.*e*., the rate of protein deposition decreases as gestation progresses). Suryawan et al. [39] reported that activation of the mammalian target of rapamycin (mTOR), and its effectors including the ribosomal protein S6 kinase (S6K1) and eIF4E-binding protein-1 (4E-BP1), which are positive regulators of protein synthesis, decreases with age in muscle. Thus, the availability of these AAs may become limited, requiring an increase in dietary intake to satisfy the growth and development of fetuses that occur during mid-gestation and late-gestation.

In the present study, we observed increases in Arg, Gly, and Tyr concentrations as gestation progressed, indicating that the demand for these AAs progressively increases throughout gestation. An insufficient supply of Arg is likely to limit the growth and development of fetal pigs during mid-gestation and late-gestation. Dietary supplementation with Arg during these periods may increase the birth weights of piglets and decrease variation in piglet birth weights [40, 41]. Notably, it has been reported that ovine fetuses have a high metabolic demand for Gly during late-gestation [42]. Additional studies have indicated that Tyr concentration increases during mid-gestation and late-gestation, and that Tyr is crucial for proper pigmentation of the skin, hair, and eyes [43]. Taken together, these findings demonstrate that fetuses need more Arg, Gly, and Tyr during mid-gestation and late-gestation to satisfy their developmental needs. Therefore, additional supplementation of these AAs during these periods may improve fetal growth and development.

The LBW fetuses exhibited higher Glu, TAA, and EAA concentrations compared with HBW fetuses at day 75 of gestation, as well as higher Tyr concentration at day 45 of gestation, indicating that the accretion of these AAs in LBW fetuses is greater than that in HBW fetuses during early-gestation or mid-gestation due to the dramatic fetal growth that occurs during late-gestation [23]. However, this result conflicts with previous reports, which indicated that transportation of AAs is decreased in IUGR fetal pigs [44, 45]. This may be because the transportation of AAs does not occur distinctly at day 45 or 75 of gestation.

FAs have remarkable metabolic and regulatory versatility in animals [36]. The fetus demands high levels of FAs, especially PUFA, for optimal growth and development. PUFA must be transported across the placenta due to the limited fetal capacity for its synthesis, especially n-3PUFA and n-6PUFA [46–49]. Fetal accretion of PUFA during the third trimester coincides with a period of substantial growth and continued organ development [50]. In the present study, C14:0, C16:0, C16:1, C18:1n9c, C18:2n6c, C18:3n3, C18:3n6, C20:0, C20:3n6, SFA, and MUFA concentrations increased as gestation progressed, indicating that the synthesis of these FAs in liver increases as gestation advances. This enables the storage of energy for fetal growth and development. Previous studies have indicated that this occurs due to growth of the liver during early-gestation [29]. As gestation progresses, C17:0, C20:4n6, C22:6n3, UFA, PUFA, n-3PUFA, and n-6PUFA concentrations decrease, as do PUFA/SFA and n-3/n-6PUFA, likely due to an inadequate supply of nutrients from the sow, decreasing the synthesis of these FAs. A previous study has also reported that levels of maternal lipids affect the FA composition of fetal tissue [51].

At days 75 and 110 of gestation, LBW fetuses exhibited the lowest C14:0, C16:1, C17:0, C18:2n6c, and MUFA concentrations and the highest C20:4n6, C22:6n3, PUFA, n-3PUFA, and n-3/n-6 PUFA concentrations, suggesting that the deposition of these FAs occurs during mid-gestation and late-gestation. FAs with the highest concentrations (including C20:4n6, C22:6n3, PUFA, n-3PUFA, and n-3/n-6 PUFA) in LBW fetuses may be more important for postnatal growth. Moreover, McNeil et al. [52] reported that the smallest fetuses do not exhibit compromised PUFA status at any stage of gestation.

## Conclusions

Maternal body composition changes as gestation progresses. We observed interaction effects between gestation period and BW on DM, CP, crude fat, Tyr, TAA, EAA, C14:0, C16:1, C17:0, C18:0, C18:2n6c, C20:4n6, C22:6n3, MUFA, PUFA, and n-6PUFA concentrations. As gestation progresses, BW increases along with DM and crude fat concentrations, while deposition of CP decreases. The most marked differences occur primarily during mid-gestation and late-gestation. LBW fetuses exhibit decreased amounts of crude fat and several FAs (including C14:0, C16:1, C17:0, and C18:2n6c) during mid-gestation and late-gestation, which may in turn affect the adaptability, growth, and development of the fetus. These findings may provide a theoretical basis for developing nutritional interventions that target fetuses with low birth weight in animals. Additional studies are needed to demonstrate the underlying mechanisms through which these effects occur during gestation.

## Supporting information

S1 Dataset Raw data of the Table 2. (XLSX)

S2 Dataset Raw data of the Table 3. (XLSX)

S3 Dataset Raw data of the Table 4. (XLS)

S4 Dataset Raw data of the Table 5. (XLS)

S5 Dataset Raw data of the Table 6. (XLS)
